# Tidal breathing parameters measured using structured light plethysmography in healthy children and those with asthma before and after bronchodilator

**DOI:** 10.14814/phy2.13168

**Published:** 2017-03-08

**Authors:** Hamzah Hmeidi, Shayan Motamedi‐Fakhr, Edward Chadwick, Francis J. Gilchrist, Warren Lenney, Richard Iles, Rachel C. Wilson, John Alexander

**Affiliations:** ^1^ Institute for Science and Technology in MedicineKeele UniversityStoke‐on‐TrentUnited Kingdom; ^2^ PneumaCare, Ltd.CambridgeshireUnited Kingdom; ^3^ University Hospitals of North MidlandsStoke‐on‐TrentUnited Kingdom; ^4^ Addenbrookes HospitalCambridgeUnited Kingdom; ^5^ Present address: Evelina London Children's HospitalLondonUnited Kingdom

**Keywords:** Asthma, bronchodilator, children, IE50_SLP_, structured light plethysmography, tidal breathing

## Abstract

Structured light plethysmography (SLP) is a light‐based, noncontact technique that measures tidal breathing by monitoring displacements of the thoracoabdominal (TA) wall. We used SLP to measure tidal breathing parameters and their within‐subject variability (v) in 30 children aged 7–16 years with asthma and abnormal spirometry (forced expiratory volume in 1 sec [FEV1] <80% predicted) during a routine clinic appointment. As part of standard care, the reversibility of airway obstruction was assessed by repeating spirometry after administration of an inhaled bronchodilator. In this study, SLP was performed before and after bronchodilator administration, and also once in 41 age‐matched controls. In the asthma group, there was a significant increase in spirometry‐assessed mean FEV1 after administration of bronchodilator. Of all measured tidal breathing parameters, the most informative was the inspiratory to expiratory TA displacement ratio (IE50_SLP_, calculated as TIF50_SLP_/TEF50_SLP_, where TIF50_SLP_ is tidal inspiratory TA displacement rate at 50% of inspiratory displacement and TEF50_SLP_ is tidal expiratory TA displacement rate at 50% of expiratory displacement). Median (m) IE50_SLP_ and its variability (vIE50_SLP_) were both higher in children with asthma (prebronchodilator) compared with healthy children (mIE50_SLP_: 1.53 vs. 1.22, *P *<* *0.001; vIE50_SLP_: 0.63 vs. 0.47, *P *<* *0.001). After administration of bronchodilators to the asthma group, mIE50_SLP_ decreased from 1.53 to 1.45 (*P *=* *0.01) and vIE50_SLP_ decreased from 0.63 to 0.60 (*P *=* *0.04). SLP‐measured tidal breathing parameters could differentiate between children with and without asthma and indicate a response to bronchodilator.

## Introduction

Assessment of respiratory function is helpful for accurate diagnosis and management of asthma (Brusasco et al. 2005; Miller et al. 2005; National Asthma Education Prevention Program 2007, Johnson and Theurer 2014; van den Wijngaart et al. 2015). Spirometry is the most commonly used technique but can be difficult or even impossible to perform in some patients due to severity of disease, extremes of age, lack of cooperation, and/or an inability to perform forced breathing maneuvers (Beydon et al. 2007). The ability to easily and noninvasively evaluate airway obstruction in young children with lung disease has the potential to improve their care.

Measurement of tidal (or “quiet”) breathing can provide useful information about respiratory function and mechanics, without requiring forced breathing maneuvers (Bates et al. 2000). Established techniques involve measurement of airflow signals with a mask or mouthpiece (e.g., pneumotachography) or assessment of signals from movement of bands placed around the thoracoabdominal (TA) wall (e.g., respiratory inductive plethysmography [RIP]) (Stick et al. 1992; Adams et al. 1993). These techniques involve contact with the patient, and the use of a mask or mouthpiece in pneumotachography can lead to alteration of tidal breathing patterns (Weissman et al. 1984; Laveneziana et al. 2015), while slippage of the transducer band may affect the data collected by RIP (Caretti et al. 1994).

Structured light plethysmography (SLP) is a noninvasive, light‐based method which enables detailed assessment of tidal breathing patterns. It measures TA wall movements by projecting a grid of light onto the anterior TA wall recorded by two digital video cameras. Average axial displacement of the light grid measures displacement over time from which tidal breathing indices can be calculated (De Boer et al. 2010; Motamedi‐Fakhr et al. 2017). It is a noncontact technique, so there is no need for the subject to use a mask, mouthpiece, or nose clip. Other than sitting still, the procedure requires minimal subject cooperation, so can be easily performed on adults and older children. In addition, with the aid of simple distraction techniques to prevent excessive subject movement, SLP has been successfully performed on children as young as 3 years old (Hmeidi et al. 2015). SLP may therefore be useful in assessing respiratory function in children and others for whom spirometry and existing tidal breathing techniques are unsuitable. For example, SLP has successfully been used to monitor tidal breathing parameters in patients who have undergone lung resection surgery (Elshafie et al. 2016).

We evaluated the use of SLP to assess tidal breathing in school‐age children with asthma and compared our findings with those from an age‐matched cohort of healthy children. We also examined the effects of bronchodilator treatment on both spirometry and tidal breathing in the group with asthma. SLP‐obtained parameters reported here include previously described and clinically used timing indices and ratios (Stocks et al. 1996; Bates et al. 2000; Baldwin et al. 2006; Lesnick and Davis 2011). Also reported are parameters obtained from the TA displacement rate signal (analogous to the flow signal in pneumotachography), regional parameters describing spatial/temporal relationships between TA regions, and within‐subject variability.

## Methods

### Study participants and design

We recruited children with asthma attending a routine outpatient clinic who demonstrated airway obstruction with abnormal spirometry, defined as forced expiratory volume in 1 sec (FEV1) <80% predicted. At our clinic, all such patients are assessed for bronchodilator reversibility. This involves repeating spirometry 15 min after administration of inhaled salbutamol (four puffs of 100 *μ*g using a metered dose inhaler and large volume spacer). Because successful performance of spirometry was necessary, the children with asthma were 7–16 years old. A cohort of healthy children of similar age and gender with no previous respiratory illnesses was also recruited. Study exclusion criteria included significant comorbidity (assessed by the pediatric clinician at screening) or chest wall abnormality, obstructive sleep apnea, any condition that in the clinician's opinion would limit the child's ability to participate, and body mass index >40 kg/m^2^. After informed consent, recruited children with asthma had two SLP assessments; the first prior to inhaled salbutamol and the second prior to repeat spirometry. Healthy children underwent one SLP assessment.

The study was approved by the UK Health Research Authority National Research Ethics Service (reference number 11/EE/00/37) and was performed at the Royal Stoke University Hospital (Stoke‐on‐Trent, UK) according to International Council for Harmonisation Guidelines for Good Clinical Practice. It is registered on ClinicalTrials.gov as part of a larger evaluation of SLP in individuals aged 2–80 years (NCT02543333). All children were enrolled between March 2014 and June 2015.

### Study devices and procedures

For each SLP assessment, tidal breathing was recorded for 5 min using an SLP device (Thora‐3Di^™^, PneumaCare, Ltd., Cambridgeshire, UK). Details of the device and how it is used are available at http://www.pneumacare.com/technology. Children were seated comfortably in a high‐backed chair as far back in the seat as possible and were asked to keep as still as they could. They either wore a close‐fitting white T‐shirt provided by the study sponsor or were assessed bare chested. A research nurse provided distraction during the procedure so that subjects breathed as naturally as possible.

The height and angle of the scanner head of the SLP device was adjusted by the researcher such that the optical axis was perpendicular to the chest wall. The midpoint of the projected grid (the cross point) was positioned at the base of the child's xiphisternum to ensure the projected area was centered on the child's TA area. The total grid pattern projected by the SLP device was adjusted to accommodate the size of each child's TA region and was set to cover an equidistant area above and below the xiphisternum from the clavicles to the anterior iliac crests. Three grid sizes with different numbers of squares were available for selection according to the child's chest size (14 × 10, 12 × 8, 10 × 6). Each square of the grid contributed equally to the signal. Sampling rate was 30 Hz, sufficient to capture the dynamics of TA wall displacement.

### Tidal breathing parameters

#### SLP assessment of tidal breathing timing indices and ratios

**Figure 1:**
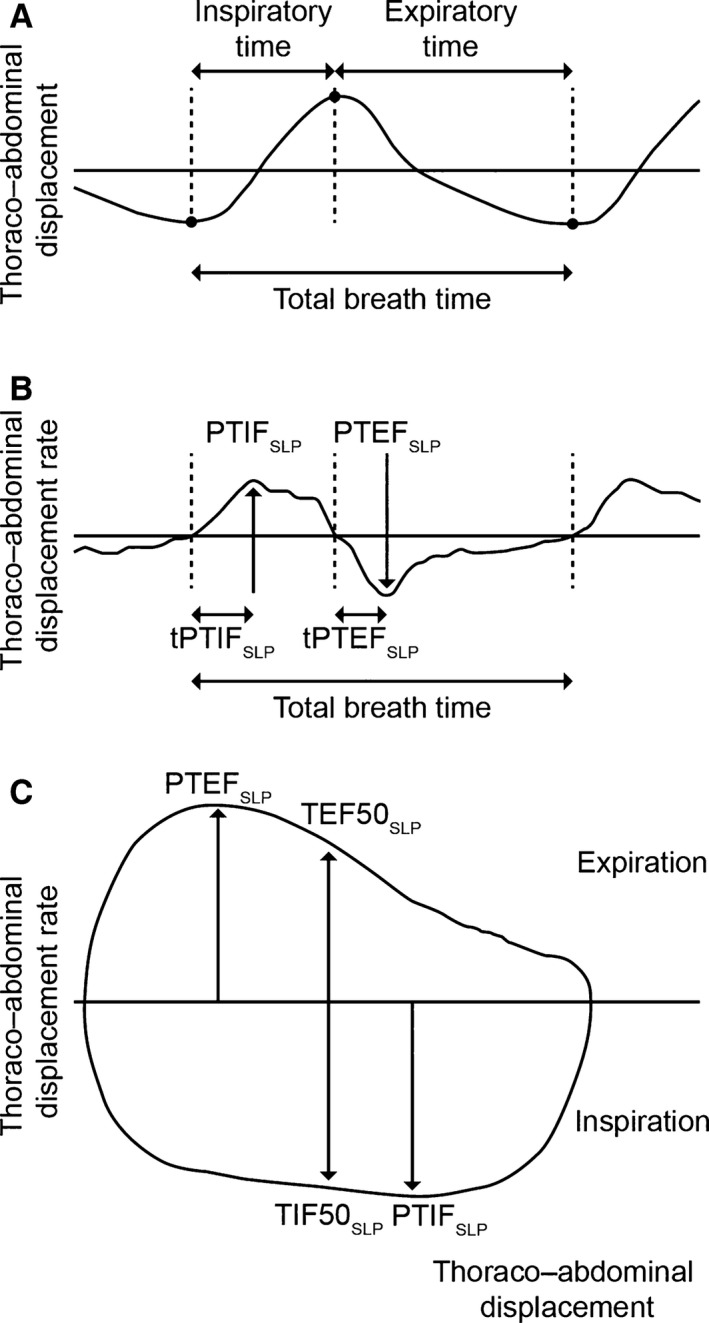
Structured light plethysmography tidal breathing traces and derived parameters. (A) Timing indices. (B) Thoracoabdominal (TA) displacement rate‐derived parameters. (C) TA displacement rate with TA displacement‐derived parameters. PTEF_SLP_, peak tidal expiratory TA displacement rate; PTIF_SLP_, peak tidal inspiratory TA displacement rate; SLP, structured light plethysmography; TA, thoracoabdominal; tPTEF_SLP_, time taken to reach peak tidal expiratory TA displacement rate; tPTIF_SLP_, time taken to reach peak tidal inspiratory TA displacement rate; TEF50_SLP_, tidal expiratory TA displacement rate at 50% of expiratory displacement; TIF50_SLP_, tidal inspiratory TA displacement rate at 50% of inspiratory displacement.

In SLP, the tidal breathing timing indices of respiratory rate (RR), inspiratory time (tI), expiratory time (tE), total breath time (tTot), and the ratios tI/tE and tI/tTot are calculated by measuring the averaged axial displacement of each intersection of a grid of light projected onto the TA wall. These timing indices correlate well with those measured by pneumotachography (Motamedi‐Fakhr et al. 2017). Figure 1A shows how the indices are calculated.

#### Tidal breathing parameters derived from flow signals

These parameters measured by pneumotachography or other methods have been well described (Stick et al. 1992; Bates et al. 2000). Tidal breathing parameters derived from plotting flow against time include peak tidal inspiratory flow (PTIF), peak tidal expiratory flow (PTEF), and time taken to reach these points (tPTIF and tPTEF). By plotting flow against volume, parameters can be generated that describe the shape of the loop. These include TEF50 (tidal expiratory flow at 50% of tidal volume) and TIF50 (tidal inspiratory flow at 50% of tidal volume). The ratio of inspiratory to expiratory flow at 50% of tidal volume (IE50) is calculated as TIF50 divided by TEF50.

#### SLP tidal breathing parameters derived from TA displacement with time signals

##### Origins and nomenclature

SLP tidal breathing parameters are derived from signals generated by TA displacement and the first derivative of TA displacement with time (i.e., TA displacement rate). SLP does not measure flow or volume, however, SLP tidal breathing parameters relating to flow are calculated in the same way as flow‐based parameters, where TA displacement is considered analogous to volume and TA displacement rate is analogous to flow. For consistency and to reflect their qualitative similarities, the same notation is used for analogous SLP parameters but with the addition of the suffix “SLP” to indicate the origin of the signal is TA displacement based.

##### TA displacement parameters (PTIF_SLP_, PTEF_SLP_, tPTIF_SLP_, tPTEF_SLP_)

Plotting TA displacement rate against time allows the following parameters to be derived: peak tidal inspiratory TA displacement rate (PTIF_SLP_), peak tidal expiratory TA displacement rate (PTEF_SLP_), time taken to reach peak tidal inspiratory TA displacement rate (tPTIF_SLP_), and time taken to reach peak tidal expiratory TA displacement rate (tPTEF_SLP_) (Fig. 1B). To correct for different respiratory rates in children, these parameters are normalized against total inspiratory and expiratory time (tPTIF_SLP_/tI and tPTEF_SLP_/tE).

##### Parameters that describe the shape of the displacement loop (TEF50_SLP_, TIF50_SLP_, IE50_SLP_)

Plotting TA displacement rate against TA displacement generates a loop analogous to a conventional tidal flow–volume loop. As with standard spirometry, parameters can be derived which describe the shape of the loop. TEF50_SLP_ is tidal expiratory TA displacement rate at 50% of expiratory displacement and TIF50_SLP_ is tidal inspiratory TA displacement rate at 50% of inspiratory displacement (Fig. 1C). IE50_SLP_ (inspiratory to expiratory TA displacement rate ratio) is TIF50_SLP_ divided by TEF50_SLP_. A validation study of SLP showed good agreement between IE50_SLP_ and IE50 measured by pneumotachography (Motamedi‐Fakhr et al. 2017).

#### SLP assessment of regional tidal breathing parameters

##### Relative contribution

**Figure 2:**
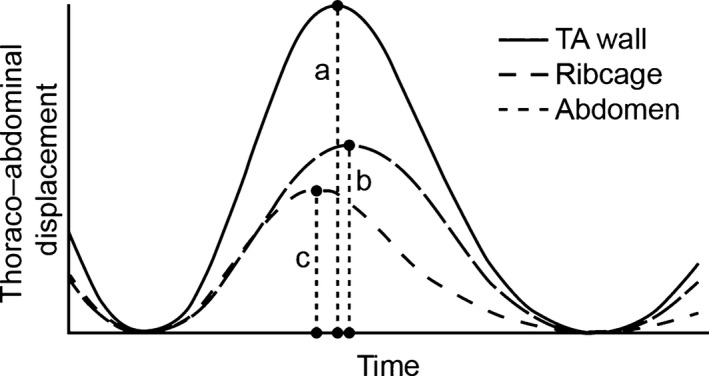
Thoracoabdominal (TA) displacement of a single breath and its thoracic and abdominal components as measured by structured light plethysmography. Dividing peak ribcage displacement (i.e., the length of dashed line b) by peak TA displacement (length of dashed line a) gives the relative thoracic contribution for the displayed breath. Dividing peak abdominal displacement (i.e., the length of dashed line c) by the length of dashed line a yields the relative abdominal contribution.

The TA region can be divided into compartments (e.g., right/left thorax and thorax/abdomen). The relative contribution of any compartment can be quantified and expressed as a percentage of total displacement. Figure 2 shows the TA displacement signal for a single respiratory cycle with its thoracic and abdominal components. To calculate the relative contribution of an arbitrary region X to an arbitrary region Y, peak‐to‐peak amplitude of each breath from region X is divided by the peak‐to‐peak amplitude of the corresponding breaths from region Y.

##### Phase

**Figure 3:**
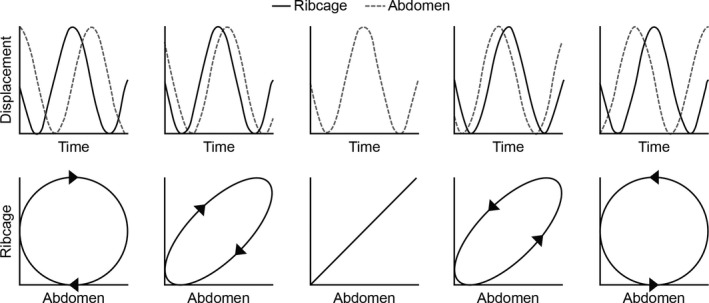
Plotting thoracoabdominal asynchrony using the method of Konno and Mead: an example. From left to right the figures show −90°, −45°, 0°, 45°, and 90° phase shifts between the hypothetical ribcage and abdomen signals. The direction of the Konno–Mead loop determines which signal is lagging behind or leading the other.

**Figure 4:**
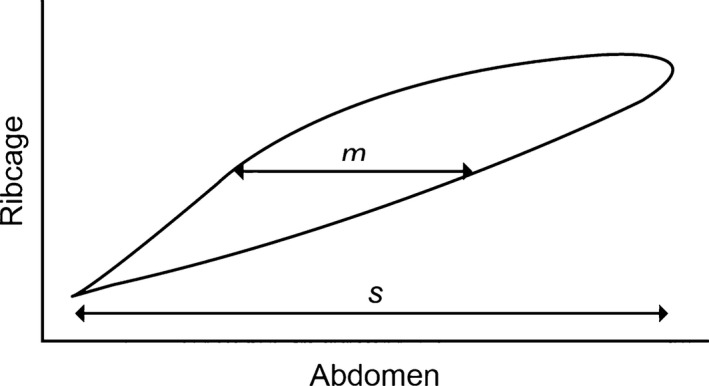
Konno–Mead loop of a single representative breath assessed by structured light plethysmography. *m* is the width of the loop at 50% of ribcage displacement and *s* is the range of abdominal displacement. Thoracoabdominal synchrony is calculated as arcsin (*m*/*sec*).

Phase describes the temporal movement of one TA region with respect to another. When there is no delay between the movement of two regions they are considered to be in synchrony. If movement of one lags behind that of the other, these regions are asynchronous. To measure asynchrony, the displacement of one can be plotted against that of the other. The shape of this graph is used to indicate the magnitude of asynchrony (Konno and Mead 1967) (Figs. 3 and 4). “Phase” is usually used only to describe thoracoabdominal asynchrony (TAA). However, SLP also allows assessment of asynchrony between the right and left compartments. Phase is quantified in degrees.

#### Variability in SLP tidal breathing parameters

Every tidal breathing parameter displays some within‐subject variability. As data are acquired over 5 min during SLP, this method allows quantification of this variability. This is achieved by calculating the interquartile range (IQR) of each parameter. IQR is a robust measure of dispersion and, unlike standard deviation, is not sensitive to the presence of outliers. This value is presented in the results with the prefix “v” to denote variability (e.g., vIE50_SLP_).

### Interpreting tidal breathing parameters: software and data analysis

PneumaView‐3D^™^ software (PneumaCare, Ltd.) allows the movement of the reconstructed TA surface to be viewed as a video. Accurate assessment of the video is essential as it may identify subtle tracking errors that are not apparent when TA displacement is plotted against time. These tracking errors can be caused by excessive creasing of the white T‐shirt or by a lack of contrast in the projected image. They cause some reconstructed points to flicker or some reconstructed surface portions to be missing. Another artifact is movement of the subject not associated with breathing, for example, a cough. This can be identified in the video as a sudden deviation of the reconstructed surface from its previous trajectory. Datasets were excluded from analysis if >50% of their respiratory cycles were affected by one or more of the above artifacts. Small breaths with peak‐to‐peak amplitudes of <25% of the median peak‐to‐peak amplitude and breaths with extremely large inspiratory and/or expiratory times were also removed as outliers.

Accepted datasets were exported by the PneumaView‐3D software. The exported data contained information on the movement of the entire TA wall, as well as regional movements. Individual breaths on all traces were automatically detected using a breath detection algorithm (Matlab, R2015b) derived from Bates et al. (2000) and Schmidt et al. (1998).

### Statistical analyses

As this study began as a pilot, and thus any findings with respect to different SLP parameters were unknown, power calculations were not carried out. For each individual SLP assessment, the median value (*m*) for each parameter over the 5‐min assessment period and its IQR (*v*) were calculated. Each SLP parameter and its variability were compared between healthy children and those with asthma (prebronchodilator) using a Mann–Whitney *U* test. The paired Wilcoxon signed‐rank test was used to assess the effect of bronchodilator in children with asthma. For all parameters showing a significant difference in these comparisons, the nonparametric common language effect size (CLES) was calculated to further describe their ability to distinguish between asthma and the healthy state, and to respond to bronchodilator. In addition, a Spearman's rank correlation was used to assess the correlation between IE50_SLP_ and lung function.

## Results

### Study population

Thirty children with asthma and 41 healthy children aged 7–16 years met the eligibility criteria and provided evaluable data for this analysis. There were no differences between children with asthma and their healthy counterparts in their age (mean ± standard deviation: 10.7 ± 2.4 and 11.2 ± 3.2 years, respectively), height (145.0 ± 17.4 and 148.0 ± 17.6 cm), or weight (41.4 ± 15.1 and 43.9 ± 17.5 kg). The numbers of males in the two groups were 17 (57%) and 21 (51%), respectively. At baseline, the airways of the children with asthma were markedly obstructed (mean FEV1 [% predicted] 68.4; mean FEV1/forced vital capacity [FVC] 69.1%).

In each group, the success rate for the SLP procedure (defined as the number of subjects providing evaluable data divided by the total number of eligible subjects) was high (asthma: 30/32 [93.8%]; healthy: 41/48 [85.4%]).

### Spirometry

After bronchodilator administration, significant increases were observed in spirometry‐obtained measures, including FEV1, FVC, and FEV1 (% predicted). FEV1/FVC (%) also significantly increased postbronchodilator but, on average, remained abnormal (mean = 76.1%), indicating airway obstruction was still present (Table 1).

**Table 1:** Comparison of spirometry parameters in children with asthma (*N* = 41) before and after bronchodilator administration

	FEV1 (L) mean±SD	FVC (L) mean±SD	FEV1/FVC (%) mean±SD	FEV1 (% predicted) mean±SD
Prebronchodilator	1.62 ± 0.64	2.36 ± 0.89	69.1 ± 10	68.4 ± 12.5
Postbronchodilator	1.93 ± 0.67	2.58 ± 0.94	76.1 ± 9.7	81.2 ± 11.2
Significance^a^	*P *<* *0.0001	*P *<* *0.01	*P *<* *0.0001	*P *<* *0.0001

FEV1, forced expiratory volume in 1 sec; FVC, forced vital capacity; SD, standard deviation.

a Significance tested using paired *t*‐test.

### Tidal breathing parameters and their within‐subject variability

Data for all median SLP‐obtained parameters and their within‐subject variability are shown in Tables 2 and 3. The median detected breaths in each SLP assessment was 82–86 and did not differ significantly in any of the comparisons performed.

**Table 2:** Comparison of tidal breathing parameters measured with SLP between children with asthma (prebronchodilator) and healthy children

	Healthy children (*N *=* *41)		Children with asthma (prebronchodilator) (*N *=* *30)		*z*‐statistic	Significance (MWU test)
	Median	IQR	Median	IQR		
Timing indices and ratios						
mRR (brpm)	19.89	7.58	20.34	5.73	0.5	0.62
vRR (brpm)	3.32	2.2	3.93	2.57	1.48	0.14
mtI (sec)	1.33	0.46	1.18	0.2	−1.9	0.06
vtI (sec)	0.27	0.17	0.24	0.14	−1.61	0.11
mtE (sec)	1.63	0.64	1.7	0.47	0.43	0.67
vtE (sec)	0.39	0.21	0.44	0.35	1.53	0.13
mtTot (sec)	3.02	1.13	2.95	0.83	−0.49	0.62
vtTot (sec)	0.53	0.32	0.56	0.44	0.72	0.47
**mtI/tE**	**0.82**	**0.16**	**0.69**	**0.1**	−**3.6**	**<0.001** b
vtI/tE	0.22	0.1	0.2	0.11	−0.36	0.72
**mtI/tTot**	**0.45**	**0.05**	**0.41**	**0.04**	−**3.61**	**<0.001** b
vtI/tTot	0.06	0.03	0.07	0.03	0.95	0.34
Displacement with time‐derived parameters						
mtPTEF_SLP_/tE	0.35	0.09	0.31	0.09	−1.81	0.07
**vtPTEF**_**SLP**_ **/tE**	**0.18**	**0.1**	**0.21**	**0.11**	**2.22**	**0.03** a
mtPTIF_SLP_/tI	0.5	0.09	0.54	0.09	1.58	0.11
vtPTIF_SLP_/tI	0.21	0.07	0.21	0.05	−0.08	0.94
**mIE50**_**SLP**_	**1.22**	**0.29**	**1.53**	**0.35**	**4.71**	**<0.001** b
**vIE50**_**SLP**_	**0.47**	**0.18**	**0.63**	**0.32**	**4.45**	**<0.001** b
Regional parameters (phase and relative contribution)						
mrCT (%)	41.96	20.04	39.18	11.3	−1.16	0.25
vrCT (%)	7.62	6.52	9.53	8.03	1.01	0.31
mHTA (degrees)	3.21	1.7	3.29	1.54	0.3	0.77
vHTA (degrees)	3.76	2.55	3.96	2.28	0.79	0.43
mTAA (degrees)	11.19	9.92	11.89	8.71	0.29	0.78
vTAA (degrees)	10.55	9.67	12.67	9.48	0.77	0.44
Number of breaths	82	26.25	84	22	0.45	0.65

For each participant, median values for each parameter over the 5‐min assessment period (denoted by the prefix *m*) and its IQR (a measure of within‐subject variability over time denoted by the prefix *v*) were calculated. Data shown are summary median and IQRs calculated by combining individual data for all participants in each group.

brpm, breaths per minute; HTA, left–right hemithoracic asynchrony; IE50_SLP_, TA inspiratory displacement rate at 50% of inspiratory displacement divided by TA expiratory displacement rate at 50% of expiratory displacement; IQR, interquartile range; MWU, Mann–Whitney *U* test; rCT, relative contribution of the thorax to each breath; RR, respiratory rate; SLP, structured light plethysmography; TA, thoracoabdominal; TAA, TA asynchrony; tE, expiratory time; tI, inspiratory time; tTot, total breath time; tPTEF_SLP_, time to reach peak tidal expiratory TA displacement rate; tPTIF_SLP_, time to reach peak tidal inspiratory TA displacement rate. Significantly different parameters are shown in bold.

a Significant with *P *<* *0.05.

b Significant with *P *<* *0.001.

**Table 3:** Comparison of tidal breathing parameters measured with SLP in children with asthma before and after bronchodilator administration

	Children with asthma (prebronchodilator) (*N *=* *30)		Children with asthma (postbronchodilator) (*N *=* *30)		*z*‐statistic	Significance (signed‐rank test)
	Median	IQR	Median	IQR		
Timing indices and ratios						
mRR (brpm)	20.34	5.73	22.16	5.91	−0.93	0.35
vRR (brpm)	3.93	2.57	4.62	2.34	−1.12	0.26
mtI (sec)	1.18	0.2	1.13	0.3	−0.85	0.40
vtI (sec)	0.24	0.14	0.23	0.1	−0.46	0.65
mtE (sec)	1.7	0.47	1.6	0.43	−1.31	0.19
vtE (sec)	0.44	0.35	0.43	0.21	−0.52	0.60
mtTot (sec)	2.95	0.83	2.71	0.77	−1.2	0.23
vtTot (sec)	0.56	0.44	0.6	0.28	−0.18	0.85
mtI/tE	0.69	0.1	0.69	0.12	−0.92	0.36
vtI/tE	0.2	0.11	0.21	0.09	−0.03	0.98
mtI/tTot	0.41	0.04	0.41	0.04	−0.89	0.37
vtI/tTot	0.07	0.03	0.07	0.03	−0.48	0.63
Displacement with time‐derived parameters						
mtPTEF_SLP_/tE	0.31	0.09	0.29	0.14	−0.57	0.57
vtPTEF_SLP_/tE	0.21	0.11	0.19	0.12	−1.39	0.16
mtPTIF_SLP_/tI	0.54	0.09	0.54	0.09	−0.66	0.51
vtPTIF_SLP_/tI	0.21	0.05	0.19	0.09	−0.75	0.45
**mIE50**_**SLP**_	**1.53**	**0.35**	**1.45**	**0.24**	−**2.44**	**0.01** a
**vIE50**_**SLP**_	**0.63**	**0.32**	**0.6**	**0.38**	−**2.05**	**0.04** a
Regional parameters (relative contribution and phase)						
mrCT (%)	39.18	11.3	39.11	12.8	−0.34	0.73
vrCT (%)	9.53	8.03	8.02	7.66	−1.61	0.11
mHTA (degrees)	3.29	1.54	3.05	1.26	−1.8	0.07
vHTA (degrees)	3.96	2.28	3.79	1.36	−1.24	0.21
mTAA (degrees)	11.89	8.71	11.73	11.44	−0.05	0.96
vTAA (degrees)	12.67	9.48	11.9	9.92	−1	0.32
Number of breaths	84	22	86	32	−0.34	0.73

For each participant, median values for each parameter over the 5‐min assessment period (denoted by the prefix *m*) and its IQR (a measure of within‐subject variability over time denoted by the prefix *v*) were calculated. Data shown are summary median and IQRs calculated by combining individual data for all participants in each group.

brpm, breaths per minute; HTA, left–right hemithoracic asynchrony; IE50_SLP_, TA inspiratory displacement rate at 50% of inspiratory displacement divided by TA expiratory displacement rate at 50% of expiratory displacement; IQR, interquartile range; MWU, Mann–Whitney *U* test; rCT, relative contribution of the thorax to each breath; RR, respiratory rate; SLP, structured light plethysmography; TA, thoracoabdominal; TAA, TA asynchrony; tE, expiratory time; tI, inspiratory time; tTot, total breath time; tPTEF_SLP_, time to reach peak tidal expiratory TA displacement rate; tPTIF_SLP_, time to reach peak tidal inspiratory TA displacement rate. Significantly different parameters are shown in bold.

a Significant with *P *<* *0.05.

**Figure 5:**
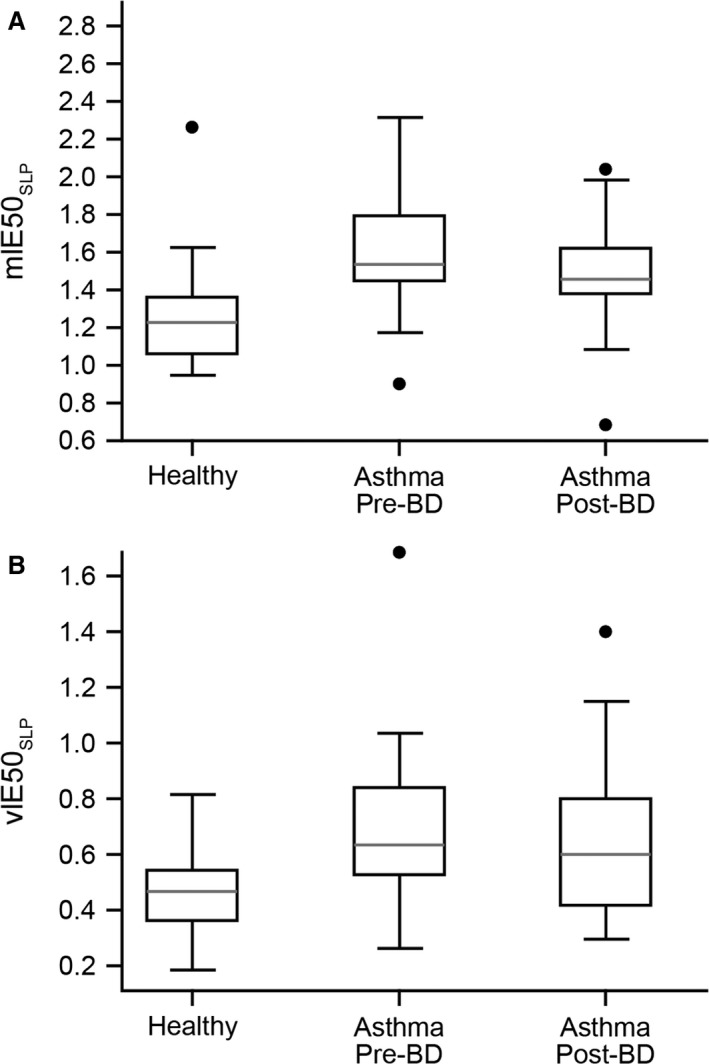
mIE50_SLP_ (A) and vIE50_SLP_ (B) in healthy children and in those with asthma (pre‐ and postbronchodilator). The gray line indicates the median value, the rectangle spans the IQR, and the black whiskers indicate the minimum and maximum values (excluding the outliers indicated by the black circles). BD, bronchodilator; IE50_SLP_, thoracoabdominal (TA) inspiratory displacement rate at 50% of inspiratory displacement divided by TA expiratory displacement rate at 50% of expiratory displacement; IQR, interquartile range; *m*, median; *v*, variability.

The inspiratory to expiratory TA displacement rate ratio (broadly analogous to inspiratory to expiratory flow ratio, i.e., IE50) and its variability were higher in children with asthma (prebronchodilator) than in the healthy children (mIE50_SLP_: 1.53 vs. 1.22, *P* < 0.001; vIE50_SLP_: 0.63 vs. 0.47, *P* < 0.001) (Table 2, Fig. 5). In the children with asthma, mIE50_SLP_ and vIE50_SLP_ decreased after bronchodilation from 1.53 to 1.45 (*P* = 0.01) and 0.63 to 0.60 (*P* = 0.04), respectively (Table 3, Fig. 5). Although both values decreased after bronchodilation, they remained higher than in the healthy group (1.45 vs. 1.22, *P* < 0.001; 0.60 vs. 0.47, *P* < 0.01) (Table 4, Fig. 5), confirming that obstruction was still present. In the subgroup of children (*n* = 16) that responded to bronchodilation (with a response defined as ≥12% increase in FEV1), mIE50_SLP_ was significantly different before and after bronchodilation (*P* = 0.038). No significant change was evident in the nonresponder group (*P* = 0.24).

**Table 4:** Comparison of tidal breathing parameters^a^ measured with SLP between children with asthma (postbronchodilator) and healthy children

	Healthy children (*n *=* *41)		Children with asthma (postbronchodilator) (*n *=* *30)		*z*‐statistic	Significance (MWU test)
	Median	IQR	Median	IQR		
**mIE50**_**SLP**_	**1.22**	**0.29**	**1.45**	**0.24**	**4.02**	**<0.001** c
**vIE50**_**SLP**_	**0.47**	**0.18**	**0.6**	**0.38**	**2.96**	**<0.01** b
**mtI/tE**	**0.82**	**0.16**	**0.69**	**0.12**	**−3.09**	**<0.01** b
**mtI/tTot**	**0.45**	**0.05**	**0.41**	**0.04**	**−3.09**	**<0.01** b
vtPTEF_SLP_/tE	0.18	0.1	0.19	0.12	0.65	0.51
Number of breaths	82	26.25	86	32	0.68	0.50

For each participant, median values for each parameter over the 5‐min assessment period (denoted by the prefix *m*) and its IQR (a measure of within‐subject variability over time denoted by the prefix *v*) were calculated. Data shown are summary median and IQRs calculated by combining individual data for all participants in each group.

IE50_SLP_, TA inspiratory displacement rate at 50% of inspiratory displacement divided by TA expiratory displacement rate at 50% of expiratory displacement; IQR, interquartile range; MWU, Mann–Whitney *U* test; SLP, structured light plethysmography; TA, thoracoabdominal; tE, expiratory time; tI, inspiratory time; tTot, total breath time; tPTEF_SLP_, time to reach peak tidal expiratory TA displacement rate. Significantly different parameters are shown in bold.

a Data are shown only for those parameters that differed between children with asthma (prebronchodilator) and healthy children (see Table 2).

b Significant with *P *<* *0.01.

c Significant with *P *<* *0.001.

**Figure 6:**
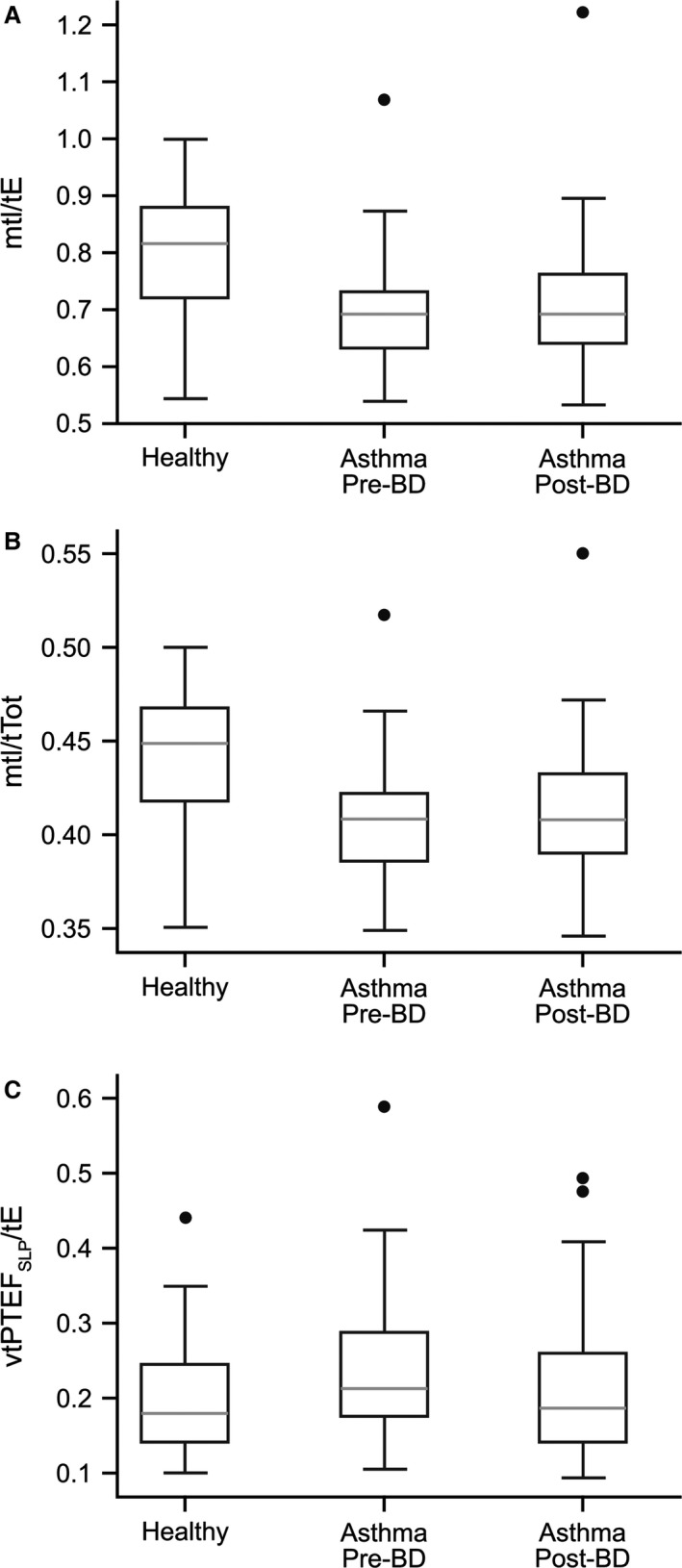
mtI/tE (A), mtI/tTot (B) and vtPTEF_SLP_/tE (C) in healthy children and in those with asthma (pre‐ and postbronchodilator). The gray line indicates the median value, the rectangle spans the IQR, and the black whiskers indicate the minimum and maximum values (excluding the outliers indicated by the black circles). BD, bronchodilator; IQR, interquartile range; *m*, median; tE, expiratory time; tI, inspiratory time; tPTEF_SLP_, time to reach peak tidal expiratory thoracoabdominal displacement rate; tTot, total breath time; *v*, variability.

Other parameters differed between the children with asthma and the healthy controls but did not change following bronchodilation. Before bronchodilation, the ratios of inspiratory to expiratory time and inspiratory to total breath time were significantly lower in children with asthma (mtI/tE, *P* < 0.001; mtI/tTot, *P* < 0.001) and the variability in the normalized time taken to reach peak tidal expiratory TA displacement rate was significantly higher (vtPTEF_SLP_/tE, *P* = 0.03) (Table 2, Fig. 6). Postbronchodilator, mtI/tE and mtI/tTot were still significantly lower (both *P* < 0.01) in children with asthma, although there was no longer a difference in vtPTEF_SLP_/tE between the two groups (*P* = 0.51) (Table 4).

**Figure 7:**
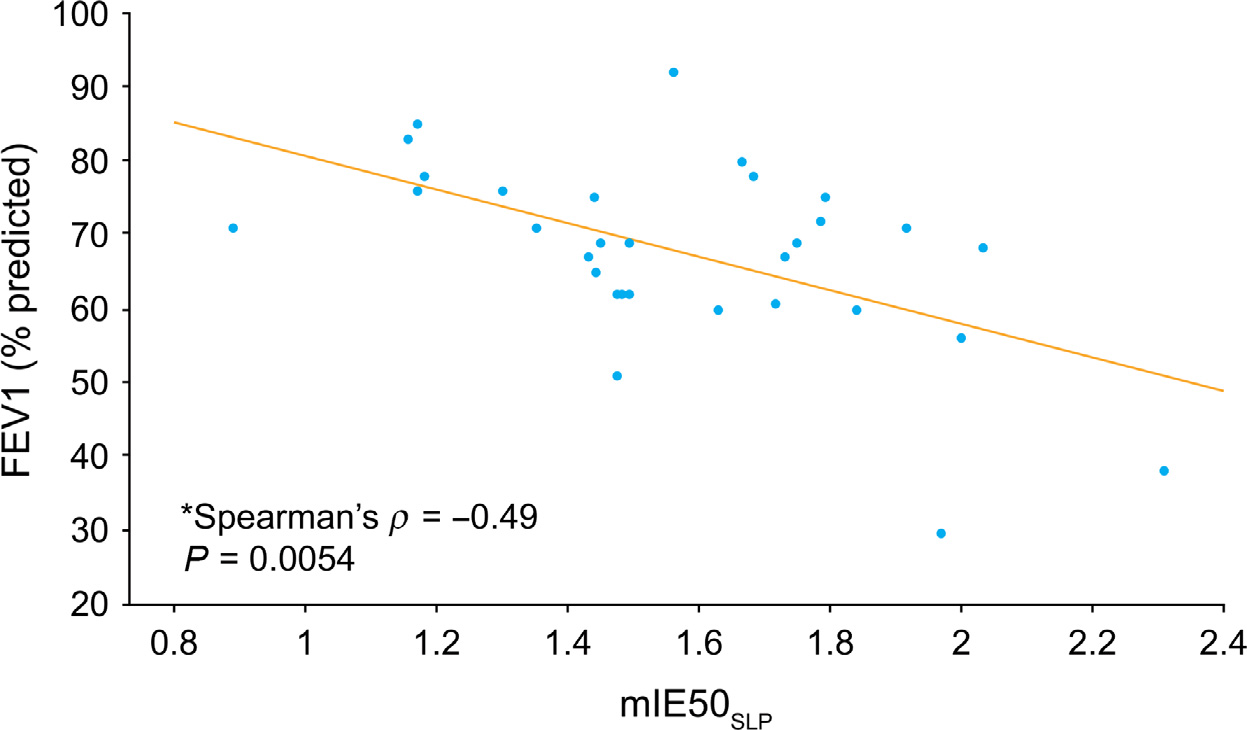
Correlation between mIE50_SLP_ and FEV1 (% predicted) in children with asthma (prebronchodilator). FEV1, forced expiratory volume in 1 sec; mIE50_SLP_, median thoracoabdominal (TA) inspiratory displacement rate at 50% of inspiratory displacement divided by TA expiratory displacement rate at 50% of expiratory displacement.

CLES evaluation demonstrated that those SLP parameters that differed significantly between the two cohorts, in particular, IE50 and its variability (CLES: 82.9% and 81.1%, respectively), could distinguish healthy children from those with asthma with a high degree of sensitivity (Table 5). Similarly, in children with asthma, these parameters could detect bronchodilator effects in the majority of cases, although they were not as sensitive as spirometry‐obtained measures (FEV1 and FEV1/FVC) (Table 5). We also performed a Spearman's rank correlation between mIE50_SLP_ and two spirometry measures in children with asthma (prebronchodilator). This test showed a correlation between mIE50_SLP_ and both FEV1 (% predicted) (−0.49, *P* = 0.0054; Fig. 7) and FEV1/FVC (−0.38, *P* = 0.034). There was no correlation between these parameters post bronchodilation. The correlation between mIE50_SLP_ and FEV1 (% predicted) remained significant in the subgroup of children who responded to bronchodilation (i.e., exhibited ≥12% increase in FEV1; *P* = 0.016), but was not significant in nonresponders (*P* = 0.25). In addition, the correlation between mIE50_SLP_ and FEV1/FVC (prebronchodilator) was not significant in either the responder (*P* = 0.08) or the nonresponder (*P* > 0.05) groups.

**Table 5:** CLES evaluation of SLP‐ and spirometry‐obtained breathing parameters

Hypothesis	CLES (%)	Interpretation
Healthy versus children with asthma^a^		
mtI/tE: lower in asthma group	75.2	In 75.2% of cases, mtI/tE was lower in asthma group
mtI/tTot: lower in asthma group	75.3	In 75.3% of cases, mtI/tTot was lower in asthma group
vtPTEF/tE: higher in asthma group	65.5	In 65.5% of cases, vtPTEF/tE was higher in asthma group
mIE50SLP: higher in asthma group	82.9	In 82.9% of cases, mIE50*SLP* was higher in asthma group
vIE50SLP: higher in asthma group	81.1	In 81.1% of cases, vIE50*SLP* was higher in the asthma group
Pre‐ versus post‐BD^b^ (children with asthma)		
*FEV1*: increases after BD	100.0	FEV1 was increased in all patients FEV1 after BD
*FEV1/FVC*: increases after BD	86.7	In 86.7% of cases, FEV1/FVC increased after BD
*mIE50SLP*: reduced after BD	70.0	In 70.0% of cases, mIE50*SLP* after BD
*vIE50SLP*: reduced after BD	73.3	In 73.3% of cases, vIE50*SLP* after BD

Median and IQR values for parameter are denoted by the prefix *m* and *v*, respectively.

BD, bronchodilator; CLES, common language effect size; FEV1, forced expiratory volume in 1 sec; FVC, forced vital capacity; IE50_SLP_, TA inspiratory displacement rate at 50% of inspiratory displacement divided by TA expiratory displacement rate at 50% of expiratory displacement; IQR, interquartile range; SLP, structured light plethysmography; tE, expiratory time; tI, inspiratory time; tTot, total breath time; tPTEF_SLP_, time to reach peak tidal expiratory TA displacement rate.

a Data are shown for parameters that significantly differed between healthy children and children with asthma (prebronchodilator) only (see Table 2). Note spirometry data were not available for healthy subjects and hence only effect sizes for SLP parameters are given.

b Data are shown for parameters that significantly differed following bronchodilator administration in children with asthma only (see Table 3).

## Discussion

Established techniques for measuring tidal breathing have limitations that restrict their use (Weissman et al. 1984; Caretti et al. 1994; Laveneziana et al. 2015). We have investigated whether SLP, a noncontact, light‐based method for measuring tidal breathing, can distinguish between children with and without asthma as well as before and after bronchodilation in those with asthma. Our results suggest that some SLP parameters can distinguish between healthy children and those with asthma. Most notably, the inspiratory to expiratory TA displacement rate ratio (mIE50_SLP_) and its within‐subject variability (vIE50_SLP_) were different between healthy subjects and asthma patients and were also sensitive to the effects of bronchodilator. This parameter is analogous to IE50, which describes the ratio of inspiratory to expiratory flow at 50% of tidal volume. Although perhaps not as sensitive as FEV1, these two SLP parameters show promise in being able to detect bronchodilator effects in a noninvasive test.

Previous studies have demonstrated that during an acute asthma attack, airway resistance increases and indices of expiratory flow such as FEV1, FEV1/FVC, peak expiratory flow, and TEF50 decreases (Papiris et al. 2002). Decreases in TEF50 have also been reported in patients similar to those recruited to our study. Using a negative expiratory pressure technique, Tauber et al. (2003) showed that TEF50 was lower in children attending an asthma outpatient clinic for a routine visit than in healthy children. We would expect that asthma‐associated decreases in TEF50 or TEF50_SLP_ would increase IE50 or IE50_SLP_. The Tauber study demonstrated that reductions in airway resistance following bronchodilator administration increased expiratory flow (Tauber et al. 2003). TEF50, however, did not return to “normal.” In our study, mIE50 decreased after bronchodilator but remained higher than in healthy children. This may indicate incomplete reversal of airway obstruction. mIE50_SLP_ has also been reported to be higher in patients with chronic obstructive pulmonary disease compared with healthy subjects (Motamedi‐Fakhr et al. 2016).

Breathing patterns are variable, allowing speech and other tasks unrelated to gas exchange to take place (Brack et al. 2002). In our study, we calculated the IQR of all parameters assessed during each SLP assessment to give a measure of within‐subject variability and showed that the variability in IE50_SLP_ (i.e., vIE50_SLP_) was higher in children with asthma than in healthy children. That asthma can affect tidal breathing variability has been known for many years. In 1985, Kuratomi et al. reported that variability in tidal volume measured by electrical impedance pneumography was significantly increased in adults experiencing an exacerbation of asthma and returned to normal after treatment. In our study, vIE50_SLP_ decreased in children with asthma after bronchodilation, but did not return to normal. Within‐subject variability in tPTEF_SLP_/tE was also higher in children with asthma (prebronchodilation) than in controls. Although vtPTEF_SLP_/tE showed no significant change after bronchodilation in children with asthma, there was no longer a significant difference between the two groups, suggesting some reduction in within‐subject variability.

We observed no differences in regional parameters in our study. For example, the relative contribution of the thorax to each breath (rCT) was similar in healthy children and in those with asthma, and there was no effect of bronchodilation. Similarly, rCT was not found to differ between patients with chronic obstructive pulmonary disease (COPD) compared with healthy subjects using SLP (Motamedi‐Fakhr et al. 2016). However, a reduction in this parameter has been observed in patients with COPD after bronchodilation (Laveneziana et al. 2014). Phase parameters describe the temporal movement of one TA region with respect to another. In children with acute asthma, synchrony between the abdomen and thorax during tidal breathing is often lost when movement of the abdomen moves ahead of the thorax. In our study, children with asthma were attending a routine outpatient clinic, were not acutely unwell, and therefore, were unlikely to display asynchrony. We are investigating whether acute exacerbations of asthma and/or their treatment affect SLP parameters, including regional ones such as rCT, TAA, and left–right hemithoracic asynchrony (HTA).

SLP is a noninvasive and noncontact technique that allows measurement of multiple consecutive breaths and has inherent advantages over established methods for assessing tidal breathing such as pneumotachography and RIP. It is important that participants remain still during SLP to avoid signal interference, although we have shown that children as young as 3 years old can be measured (Hmeidi et al. 2015). Operators should also be aware of the possibility of subtle tracking errors that may not be reflected in the respiratory trace. As described in the Methods section, such errors can be detected and data excluded.

Multiple statistical comparisons were made during our study. The risk of some statistically significant results occurring by chance was therefore considered. Applying the Bonferroni correction method for our 24 comparisons produced a *P* < 0.002 (0.05/24). This method, however, assumes that all comparisons are independent. This is not the case as many of the SLP‐measured parameters are correlated. At least some changes in SLP parameters appear to have a firm physiological basis and/or are corroborated by previous studies. CLES evaluation also supported the findings of the initial statistical comparisons.

A prerequisite for recruitment was confirmation of airway obstruction using spirometry. This was necessary to provide a recognized “standard” for the presence of, and changes in, airways obstruction on which to base SLP comparisons. Thus, enrollment of younger children who might benefit most from this technique was effectively excluded. Our other study in children with acute asthma has recently been completed and may provide useful information on this patient population.

## Conclusion

We have shown that SLP – a noncontact and noninvasive method for measuring tidal breathing – can differentiate between children with and without airway obstruction and may identify responses to bronchodilator. Further research to confirm these observations is underway.

## Endnotes

At the request of the authors, readers are herein alerted to the fact that additional materials related to this article may be found at the institutional website of one of the authors, which at the time of publication they indicate is: http://www.pneumacare.com/technology. These materials are not a part of this manuscript and have not undergone peer review by *Physiological Reports*. The editors take no responsibility for these materials, for the website address, or for any links to or from it.

## Conflict of Interest

R. I. is a shareholder of PneumaCare, Ltd. and was also a part‐time paid medical advisor to PneumaCare, Ltd. at the time of the study. R. C. W. and S. M. F. are employees of and have share options for PneumaCare, Ltd. W. L. is employed part‐time as a pediatric respiratory advisor to GlaxoSmithKline. E. C., H. H., J. A., and F. J. G. have declared no conflicts of interest, financial, or otherwise.
